# Extended lower paratracheal lymph node resection during esophagectomy for cancer – safety and necessity

**DOI:** 10.1186/s12885-022-09667-1

**Published:** 2022-05-24

**Authors:** C. Mann, F. Berlth, E. Hadzijusufovic, E. Tagkalos, E. Uzun, C. Codony, H. Lang, P. P. Grimminger

**Affiliations:** 1 Department of General, Visceral and Transplantation Surgery, University Medical Center Mainz, Langenbeckstr. 1, 55131 Mainz, Germany; 2 Department of Surgery, Hospital Universitari Josep Trueta, Girona, Spain

**Keywords:** Esophageal cancer, Esophagectomy, Lymphadenectomy, Paratracheal lymph nodes, Lymph node metastases

## Abstract

**Background:**

The ideal extent of lymphadenectomy (LAD) in esophageal oncological surgery is debated. There is no evidence for improved survival after standardized paratracheal lymph node resection performing oncological esophagectomy. Lymph nodes from the lower paratracheal station are not standardly resected during 2-field Ivor-Lewis esophagectomy for esophageal cancer. The objective of this study was to evaluate the impact of lower paratracheal lymph node (LPL) resection on perioperative outcome during esophagectomy for cancer and analyze its relevance.

**Methods:**

Retrospectively, we identified 200 consecutive patients operated in our center for esophageal cancer from January 2017 – December 2019. Patients with and without lower paratracheal LAD were compared regarding demographic data, tumor characteristics, operative details, postoperative complications, tumor recurrence and overall survival.

**Results:**

103 out of 200 patients received lower paratracheal lymph node resection. On average, five lymph nodes were resected in the paratracheal region and cancer infiltration was found in two patients. Those two patients suffered from neuroendocrine carcinoma and melanoma respectively. Cases with lower paratracheal lymph node yield had significantly less overall complicated procedures (*p =* 0.026). Regarding overall survival and recurrence rate no significant difference could be detected between both groups (*p =* 0.168 and 0.371 respectively).

**Conclusion:**

The resection of lower paratracheal lymph nodes during esophagectomy remains debatable for distal squamous cell carcinoma or adenocarcinoma of the esophagus. Tumor infiltration was only found in rare cancer entities. Since resection can be performed safely, we recommend LPL resection on demand.

## Introduction

With worldwide 436,000 deaths in 2017 and an increase in the mortality rate of 13% from 2007 to 2017 esophageal cancer is a threatening oncological burden [1]. Despite advanced multimodal treatment strategies, prognosis remains poor. The interdisciplinary therapeutic approach - including surgery, chemotherapy, and radiation - achieves 5-years-survival rates across all stages up to 2.8–50% [2]. Regarding disease free and overall survival, as well as recurrence free survival, surgery is an integral part of therapy. Thereby, extended lymphadenectomy seems to be crucial [3, 4]. Due to a great number of lymphatic routes in the submucosal layer of the esophagus, cancer cells, even from superficial tumors, can spread easily and fast. They follow the lymphatic routes longitudinally along the submucosal plexus, intermittently pierce through the muscularis mucosa and drain into local lymph nodes or directly into the thoracic duct without respecting segmental drainage areas [5]. Therefore, lymph node metastases occur frequently irrespective of the tumor location, making a thorough lymphadenectomy inevitable for possible cure. However, extended lymphadenectomy with radical dissection around vital and delicate structures - like the thoracic duct, the bronchi, the trachea, and the pleura - can lead to major postoperative complications. These complications contribute to the already high mortality rate after surgical esophagectomy [6]. Therefore, oncological necessity and surgical radicality need to be thoroughly pondered. The nowadays commonly performed esophagectomy in Europe consists of a thoraco-abdominal approach with two field-lymphadenectomy (2FD). In contrast, most Eastern studies favor an extended three field-lymphadenectomy (3FD) - including bilateral cervical, mediastinal, and abdominal regions - justified by improved overall survival [7]. However, since 3FD is accompanied by a higher complication rate, definite conclusions have not been drawn yet [8].

**Fig. 1:**
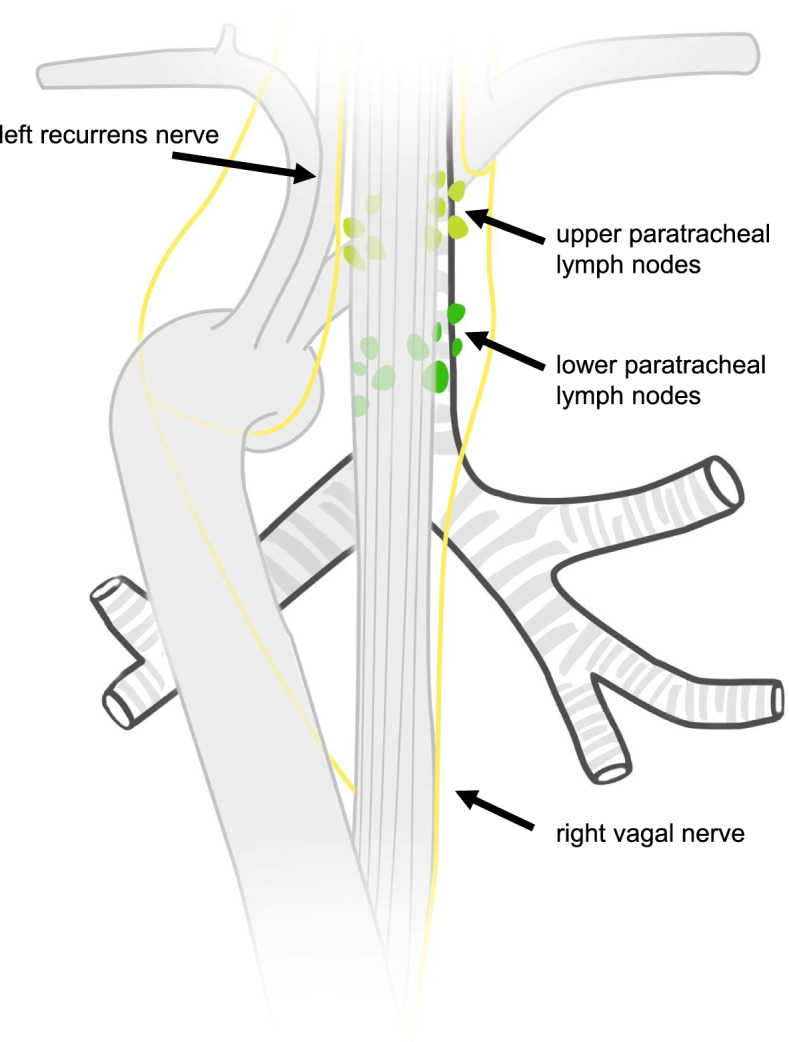
Dorsal view on trachea with location of upper and lower paratracheal lymph nodes (light and dark green)

In particular, the necessity of lower paratracheal lymph node resection remains unclear. As mentioned above, the esophagus drains its lymph to adjacent lymph nodes and the thoracic duct, which accompanies the esophagus on its dorsal right side heading to the left angulus venosus [9]. Shiozaki et al. described the left and right recurrent nerve chain as the main drainage area cranial from the tracheal bifurcation [10]. The lymph nodes in the lower paratracheal region lying ventral of the esophagus might be less affected by metastasis. Their resection is part of an extended mediastinal lymph node dissection during 2FD (Fig. 1). Due to the improvement of technological support in minimal invasive surgery such as robot assistance, accessing to these regions and dissecting without risking collateral damage has become simplified. Thus, although lying between vulnerable structures, lower paratracheal lymph nodes can be resected on a more frequent basis. The aim of this study was to evaluate the perioperative safety of such an extended lymph node yield, with so far unknown oncological benefit.

## Methods

### Definition of lymph node field

So far, the exact distribution pattern of lymph node metastases from esophageal cancer is unknown. In order to achieve comparable data, we followed the definition of the ongoing TIGER-study for lymphadenectomy in our institution [11]. In this classification the lower paratracheal region is part of the extended 2FD (Fig. 1, dark green).

**Fig. 2:**
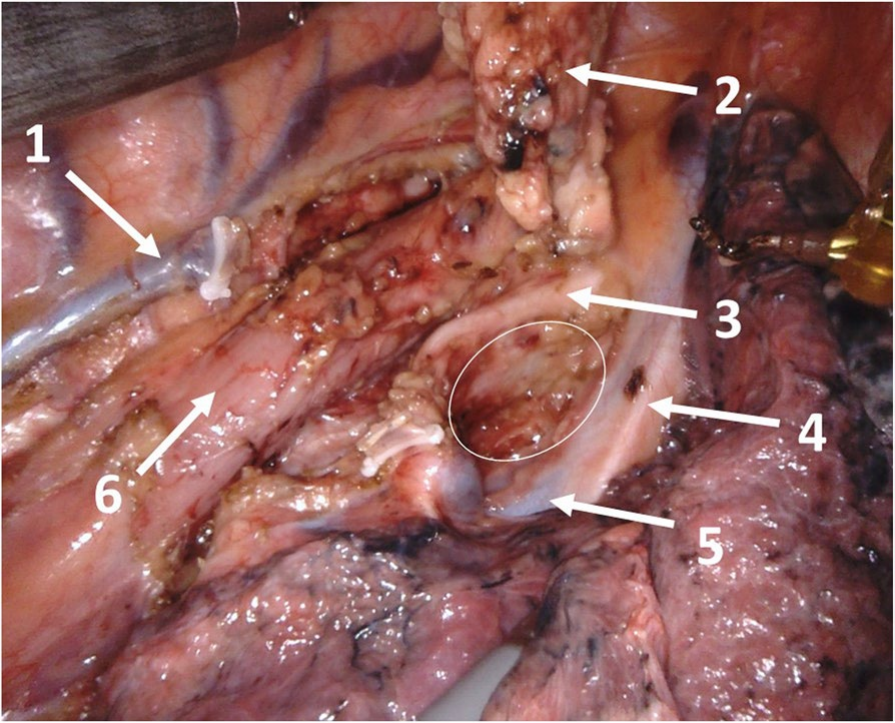
Operative site (view from right dorsal side) after right LPL resection. 1 = azygos vein, 2 = LPL specimen, 3 = trachea, 4 = phrenic nerve, 5 = superior vena cava, 6 = esophagus, Circle = LPL region after LPL resection

We defined the upper paratracheal region (light green) as lymph nodes located along the left and right recurrent laryngeal nerve in the mediastinum. In this study only the lower paratracheal lymph nodes were analyzed. In our definition pretracheal lymph nodes, paraesophageal lymph nodes, recurrent lymph nodes as well as tracheobronchial lymph nodes were not part of the LPL region. Figure 2 shows the intraoperative site after LPL resection using robotic assistance.

### Patients

We analyzed retrospectively 200 patients from January 2017 – December 2019 who underwent either total minimally invasive or robot assisted minimally invasive esophagectomy in our center, University medical center of the Johannes-Gutenberg-University Mainz, Germany. Included were all patients operated for cancer at any stage, tumor location, histological entity, or possible application of neoadjuvant therapy. Patients with distant metastases (of adenocarcinoma or squamous cell carcinoma) or benign underlying disease were excluded since tumor infiltration of LPL was of the main interest in this study. Patients were allocated in two groups depending on the extent of lymph node yield in the paratracheal region. Data was collected in both groups regarding patients’ demographics (age, gender, BMI, comorbidity, ASA score), tumor characteristics (preoperative lymph node status, tumor type and location, possible neoadjuvant therapy), operative details (type of approach, operation time in minutes) as well as postoperative data (postoperative complications, intensive care unit stay (ICU) and hospital stay, readmission to ICU) and survival (30-day mortality, 90-day mortality, overall survival, metastasis and recurrence).

### Surgical procedure

Esophagectomy was performed as a totally minimally invasive approach conducted in two stages [12–15]. In 71,5% of the cases robotic assistance was used. In the first stage, laparoscopic gastric mobilization, lymphadenectomy at the lesser curvature and creation of the gastric conduit was performed in supine position of the patient. In the second stage, the patient was placed to the left semiprone position for ideal access to the dorsal mediastinum. The esophagus was mobilized and the lymph nodes from the paraesophageal, pulmonary ligament, subcarinal as well as aortopulmonary station were dissected and removed preferable en bloc with the specimen. The lower paratracheal lymph node station was resected depending on the surgeon’s judgment. The decision was made based on two major reasons: advanced tumor stage (T3) and preclinical or intraoperatively suspicious nodes. Indeed, the majority (82%) of patients receiving LPL resection were operated using robotic assistance. An esophagogastric anastomosis was created intrathoracically with a circular end-to-end anastomotic stapler through a mini-thoracotomy. All surgeries were performed by the same surgeon.

### Postoperative complications

Complicated procedures were defined as procedures with any postoperative complication such as: postoperative bleeding, pulmonary complication, chylothorax, anastomotic leakage, neurological complication, laryngeal nerve paralysis, cardiac complication, sepsis, wound infection or delayed gastric emptying. Postoperative complications were graded according to the Clavien-Dindo-Classification as minor (1–2) or major (3–5) complications. Pulmonary complications (e.g., pneumonia, pleural effusion) and anastomotic leakage were analyzed separately. 30- and 90-day mortality was analyzed and compared. Complications were collected in a prospective institutional database. Complications were classified according to the recommendations of the consensus groups [16].

### Statistical analysis

Data were reported as median or mean ± SD. All data were tested for normal distribution using the Shapiro-Wilk test. Normal distributed data were compared using the Student’s T-test. Comparison between not normal distributed groups was made using a Mann-Whitney-test. Categorical variables were analyzed using the Chi-squared test. Overall survival was depicted using the log rank test. Statistical analysis was performed using standardized biomedical software (SPSS Version 27). Differences were considered significant at *p <* 0.05.

## Results

Histopathologically, 143 patients had an adenocarcinoma, 53 patients a squamous cell carcinoma, two patients a neuroendocrine carcinoma, and one suffered from melanoma of the esophagus. The median number of harvested lymph nodes in all patients was 30. In 103 of 200 cases (51%) lower paratracheal lymph node resection was performed, the rest (97 cases, 49%) did not receive lower paratracheal lymph nodes resection (Table 1). Significantly more total lymph nodes were resected in the LPL group (median of 35 vs. 25). Thereby, a median of 5 lymph nodes were harvested in the lower paratracheal region with histopathological cancer positivity in 2 patients. Those two patients suffered from melanoma with an esophageal metastasis and a neuroendocrine tumor of the esophagus.

**Table 1:** Clinicopathological parameters and comparison between both groups

	LPL resection (*n =* 103)	No LPL resection (*n =* 97)	*p*-value
**Total number of lymph nodes resected per patient (median)**	35	25	0.001
**Total number of LPL resected (median-range)**	5 (1–23)	0	
**Age (y) (mean – SD)**	63 (10.3)	65 (10.9)	0.266
**Gender (n (%))**			
M	81 (79)	80 (83)	
F	21 (21)	17 (17)	
**BMI (kg / m**^**2**^**) (median – range)**	24.8 (14.9–46)	25.7 (13.8–36)	0.295
**Co-morbidity (n (%))**			0.117
No comorbidity	30 (29)	19 (20)	
Comorbidity	73 (71)	78 (80)	
**ASA score (n (%))**			0.592
2	48 (47)	40 (41)	
3	52 (50)	52 (54)	
4	3 (3)	5 (5)	
**Preoperative Lymph Node Status (cN) (n (%))**			0.568
cN0	26 (25)	33 (34)	
cN1	61 (59)	49 (51)	
cN2	12 (12)	11 (12)	
cN3	1 (1)	0 (0)	
cNx	3 (3)	3 (3)	
**Tumor depth (n (%))**			0,683
T0	18 (17.5)	19 (19.6)	
T1	13 (12.6)	20 (20.6)	
T2	15 (14.6)	11 (11.3)	
T3	54 (52.4)	45 (46.4)	
T4	3 (2.9)	2 (2.1)	
**Tumor location (n (%))**			0.728
Upper esophageal	3 (3)	2 (2)	
Middle esophageal	19 (18)	13 (13)	
Lower esophageal	55 (54)	58 (60)	
Cardia	26 (25)	24 (25)	
**Tumor type (n (%))**			0.681
Adenocarcinoma	72 (70)	71 (73)	
Squamous cell carcinoma	29 (28)	24 (25)	
Melanoma	1 (1)	0 (0)	
Neuro-endocrine	1 (1)	1 (1)	
No viable tumor cells	0 (0)	1 (1)	
**Neoadjuvant treatment (n (%))**			0.051
No therapy	16 (16)	26 (27)	
Therapy	87 (84)	71 (73)	

Data regarding lymph node resection, patient demographics and tumor characteristics, *BMI* Body-mass-index, *ASA* American Society of Anaesthesiologists

### Patient demographics and tumor characteristics

There was no significant difference in the two groups in terms of age, gender, BMI, comorbidity, or American Society of Anesthesiology classification. Patients without comorbidities tended to receive lower paratracheal lymph node resection more frequently, however this did not reach statistical significance.

Lower paratracheal lymph nodes were harvested irrespective of the preoperative lymph node status in the CT-Scan, the tumor location, and the type of tumor. However, from the three patients suffering from cancer other than adenocarcinoma or squamous cell carcinoma, two received lymph node resection in the lower paratracheal region. 80,2% of all tumors were located at the lower esophagus or cardia-region. Patients with neoadjuvant treatment tended to receive lower paratracheal lymph node resection on a more frequent basis than patients not pretreated. Neoadjuvant treatment was conducted for patients with a pre-treatment staging >T2 or N+, diagnosed by CT-Scan and endoscopic ultrasound. However, this difference did not reach statistical significance. Comparing only adenocarcinoma and squamous cell carcinoma both received lower paratracheal lymph node resection at a similar rate (50.3%, respectively 54.7%). Distribution between adenocarcinoma and squamous cell carcinoma was statistically equal in both groups. (70 and 28% vs. 73 and 25%) (Table 1).

### Operative and postoperative characteristics

The patients receiving a resection in the lower paratracheal stations were significantly more often operated with robotic assistance (*p <* 0.001), with a trend towards shorter operation time. However, time difference arises from the abdominal part and is therefore not likely caused by the extended lymphadenectomy. There was no difference regarding intraoperative complications in both groups.

Interestingly, cases with lower paratracheal lymph node yield had significantly less overall complicated procedures (*p =* 0.026) according to the Esophageal Complications Consensus Group (ECCG) definitions [6]. Discriminating between major and minor complications according to the Clavien-Dindo-classification, the paratracheal harvested group resulted in significantly more minor and less major complications. No differences were found regarding pulmonary complications rate or anastomotic leakage rate. Concomitant with the lower complication rate, cases including lower paratracheal lymph node resection had a shorter median hospital stay (*p <* 0.05) and a trend towards less readmissions to intensive care unit (*p =* 0,097). Overall stay at ICU showed no significant difference between both groups (Table 2).

**Table 2:** Comparison of operative and postoperative data between both groups

	LPL resection (*n =* 103)	No LPL resection (*n =* 97)	*p*-value
**Approach (n (%))**			0.001
MIE	19 (18)	38 (39)	
RAMIE	84 (82)	59 (61)	
**Operating time (min)(SD)**			
Total operating time	377.4 (54.8)	394.6 (74.5)	0.075
Thoracic part	205 (35.2)	203 (53.4)	0.677
**Uncomplicated procedures (n (%))**	68 (66)	49 (51)	0.026
**Complicated procedures (n (%))**	35 (34)	48 (49)	
**Clavien-Dindo Classification**	(*n =* 35)	(*n =* 48)	
Minor Complication (Clavien-Dindo 1–2)	20 (57)	16 (33)	0.031
Major Complication (Clavien-Dindo 3–5)	15 (43)	32 (67)	
**Pulmonary complications (n (%))**	23 (22)	28 (29)	0.289
**Anastomotic leakage (n (%))**	9 (9)	14 (14)	0.207
**Intensive care unit (ICU) stay (days) (median – range)**	1 (0–115)	2 (0–84)	0.677
**Readmission ICU (n (%))**	9 (9)	16 (17)	0.097
**Hospital stay (days) (median – range)**	11 (7–115)	12 (7–91)	0.005
**Readmission in 30 days after discharge (n (%))**	14 (14)	15 (16)	0.757

Operative and postoperative details, *MIE* Minimal invasive esophagectomy, *RAMIE* Robotic assisted minimal invasive esophagectomy, *SD* Standard deviation

### Survival

**Fig. 3:**
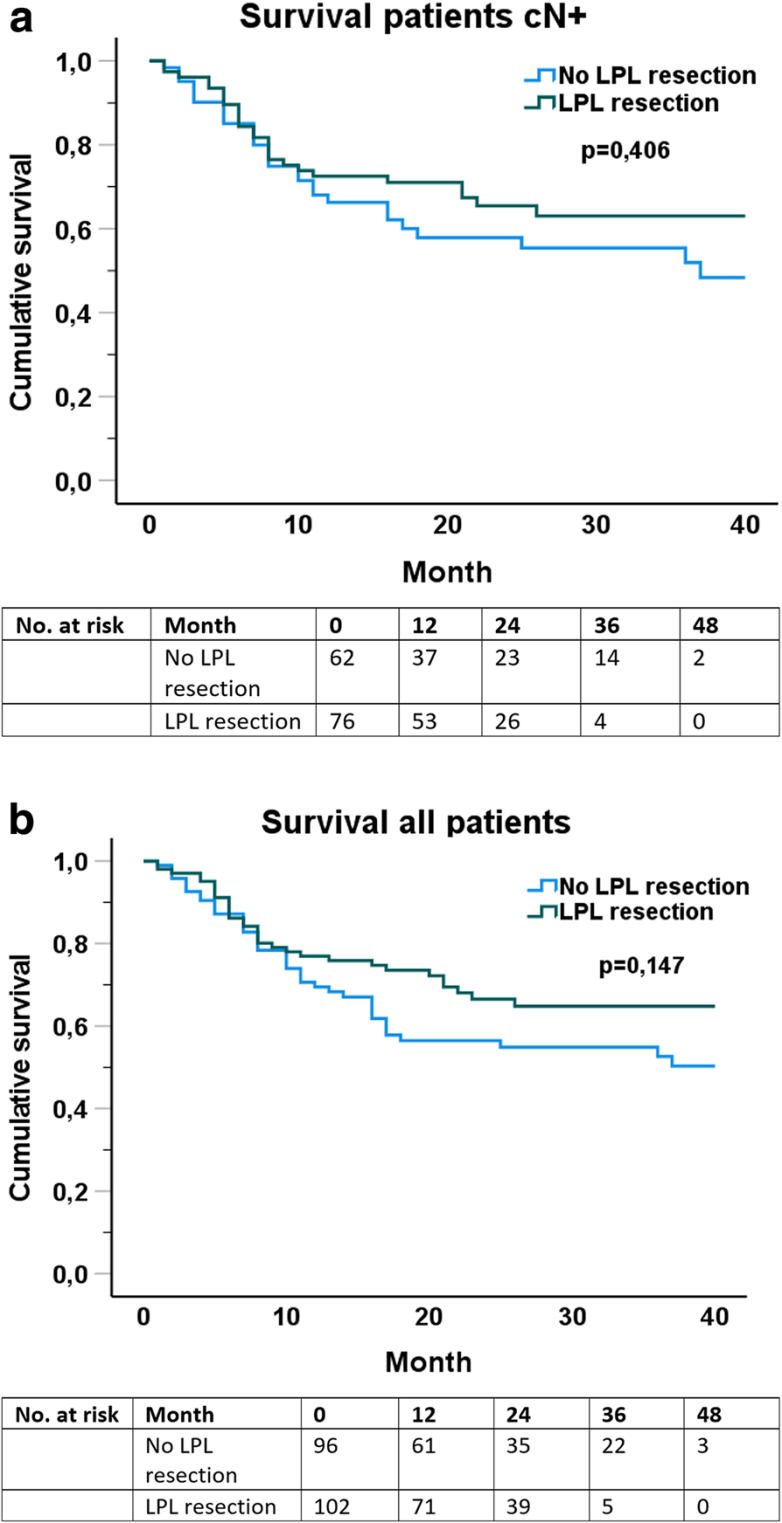
Survival of patients with positive preoperative lymph node status (**a**) and all patients combined (**b**)

Resection of paratracheal lymph nodes had no significant effect on either 30- or 90-day intrahospital mortality (*p =* 0,943, respectively 0,919). There was no difference between both groups regarding occurrence of metastasis and tumor recurrence. Regarding overall survival we analyzed patients with a preoperatively nodal positive tumor stage (cN+) separately. The nodal positive patients as well as all patients showed a trend towards better survival with LPL resected. However, this did not reach statistical significance (Fig. 3, *p =* 0,406 and 0,147) (Table 3). Median survival follow-up was 27 months.

**Table 3:** Survival of both groups

	LPL resection (*n =* 103)	No LPL resection (*n =* 97)	*p*-value
**30 day mortality (n (%))**	2 (2)	2 (2)	0.943
**90 day mortality (n (%))**	4 (4)	4 (4)	0.919
**Overall survival (month) (mean - SD)**	32.1 (1.48)	29.9 (2.1)	0.147
**Metastasis or recurrence (n (%))**	40 (38.8)	37 (38.1)	0.440

Mortality rate, survival, and recurrence rate, *SD* Standard deviation

## Discussion

The required extent of lymphadenectomy during esophagectomy for cancer remains a controversial topic [17, 18]. In current literature the benefit of an increased lymph node yield on overall survival, with at least 15–23 resected lymph nodes, has been proven [3]. However, the explicit location of affected lymph nodes is still under debate. A distribution pattern of metastatic lymphatic spread in both adenocarcinoma and squamous cell carcinoma identified by Hagens et al. in a systematic review, found metastases to cervical, thoracic, and abdominal lymph node stations, regardless of the primary tumor location [11]. Even though higher accumulations of lymph node metastases are located nearby the primary tumor (depending on tumor location), distribution percentages differ and distant nodal metastasis as well as skip metastasis (metastasis infiltrating more distant lymph nodes without affecting adjacent nodes) are frequently seen in both esophageal adenocarcinoma and squamous cell carcinoma [19, 20].

Resection of these distant lymph node metastases, e.g. in the proximal mediastinal field, is not only beneficial for oncological radicality. Pathologically, systematic lymph node resection enables an exact postoperative tumor staging, important for further therapy decisions [21–23]. Additionally, different studies described reduced hazard of death with an increasing number of resected and examined nodes [24, 25]. Despite these benefits, possible complications and harms should also be considered. In order to find an accurate statement for necessary lymph node resection, a separate assessment of each lymph node station is required. The lower paratracheal lymph node station (station 7 according to TIGER-study) turned out to be the most striking station of our data. The resection of the paratracheal lymph nodes is considered as the extension of standard 2FD, which consist of the abdominal lymph node stations as well as a complete dissection of the middle and lower mediastinal nodes, including the paraesophageal, pulmonary ligament, subcarinal, and aortopulmonary window nodes.

We found no positive lymph node in the lower paratracheal region of all 101 patients with adenocarcinoma or squamous cell carcinoma of the esophagus undergoing LPL resection. In fact, the only two patients with positivity in this region suffered from different histopathological entities (melanoma and neuroendocrine tumor). This is the main finding of this study.

The resection of LPL had no significant effect on either 30- and 90-day mortality or tumor recurrence. However, overall survival (Fig. 3) showed a trend towards better survival for LPL resected patients. Since tumor infiltration of the LPL region was only found in two cases the resected LPL region might not be the underlying cause. Interestingly, total lymph node harvest was significantly higher in the LPL group with 35 vs. 25 lymph nodes, with only five resected lymph nodes in the paratracheal region on average. One could argue, when LPL resection was conducted, an overall more thorough LAD was performed, leading to improved survival. Additionally, the lower complications rate of the patients receiving LPL resection might be an important factor for an improvement of long-term survival independent of oncological reasons. These arguments show that LPL resection can be performed without increased morbidity or mortality.

A large cohort study by Harada et al., conducted in the United States, was the first to investigate paratracheal lymph node metastasis from adenocarcinoma of the esophagus [26]. Excluding the cases with initial lymph node metastases in the paratracheal region, 6,5% of the analyzed patients who did not have received LPL suffered from positive paratracheal lymph node recurrence later. However, their definition of the paratracheal regions differed strongly from ours. According to the Japanese Classification of Esophageal Cancer, 11th Edition [27], they included upper thoracic paraesophageal lymph nodes, cervical paraesophageal lymph nodes, recurrent nerve lymph nodes and left tracheobronchial lymph nodes.

There are limitations to this study. Firstly, it is a retrospective study, lacking a randomization. The decision for or against paratracheal resection was made by the surgeon. Thus, pretreated patients (chemotherapy or chemoradiation) tended to receive paratracheal lymph node resection more frequently than patients not pretreated (without reaching significance *p =* 0.051) – most likely due to the surgeons’ expectations for a more extended underlying disease and the higher probability for a locally advanced tumor (T3–4). However, there was found no difference between both groups regarding occurrence of metastasis and tumor recurrence. Additionally, during operations with robotic assistance, paratracheal stations were resected at a significantly higher rate. The simplification of meticulous dissection between delicate structures allows greater radicality. In fact, the trend towards faster procedures when including paratracheal lymph node resection hints at the same point: easier procedures are more often combined with extended lymph node resection. Supporting this theory, patients not receiving paratracheal lymph node resection had significantly more complicated procedures (*p =* 0.026) and more major complications according to Clavien-Dindo-classification. Additionally, in 2017 only 22% of the patients received LPL resection, the same year robotic assistance was introduced in our clinic. Some part of the higher complication rate in the non-dissecting group might be due to the learning curve after introduction of robotic assistance. In order to eliminate these bias mistakes, a prospective study with randomization is required.

Based on the presented data, we do not perform standardized resection of the lower paratracheal lymph node station when operating adenocarcinoma and squamous cell carcinoma of the distal esophagus in our institution. Since modern technologies enable safe dissection and no increase of morbidity due to the paratracheal dissection, LPL should always be considered in rare cancer entities or on demand.

## Conclusion

In summary, the current report shows that tumor infiltration of LPL during esophagectomy for distal squamous cell carcinoma or adenocarcinoma of the esophagus is rare. We cannot recommend LPL resection by default. Resection of LPL caused no increased postoperative morbidity and was accompanied by a higher total number of lymph nodes harvested. Further accumulation of data is required for explicit conclusions on necessary dissection of each lymph node station since distribution of metastasis for both adenocarcinoma and squamous cell carcinoma is very heterogeneous. Irrespective of that, resection of clinically or intraoperative conspicuous lymph nodes should be decided individually.

## Data Availability

The datasets analysed during the current study are not publicly available due to future analysis and publication plans, but are available from the corresponding author on reasonable request.
